# Genomic analysis of *Salmonella enterica* serotype Paratyphi A during an outbreak in Cambodia, 2013–2015

**DOI:** 10.1099/mgen.0.000092

**Published:** 2016-11-30

**Authors:** Laura Maria Francisca Kuijpers, Simon Le Hello, Nizar Fawal, Laetitia Fabre, Mathieu Tourdjman, Muriel Dufour, Dara Sar, Chun Kham, Thong Phe, Erika Vlieghe, Christiane Bouchier, Jan Jacobs, François-Xavier Weill

**Affiliations:** ^1^​ Department of Clinical Sciences, Institute of Tropical Medicine, Antwerp, Belgium; ^2^​ Department of Microbiology and Immunology, KU Leuven, Leuven, Belgium; ^3^​ Institut Pasteur, Unité des Bactéries Pathogènes Entériques, Centre National de Référence des Escherichia coli, Shigella et Salmonella, Paris, France; ^4^​ Santé Publique France, Direction des Maladies Infectieuses, Saint-Maurice, France; ^5^​ Institute of Environmental Science and Research Limited, NCBID, Wallaceville, New Zealand; ^6^​ Sihanouk Hospital Centre of HOPE, Phnom Penh, Cambodia; ^7^​ Department of Tropical Diseases, University Hospital Antwerp, Antwerp, Belgium; ^8^​ Institut Pasteur, Plate-forme Génomique (PF1), Paris, France

**Keywords:** Cambodia, *Salmonella* Paratyphi A, whole genome sequencing, resistance

## Abstract

In 2013, an unusual increase in the number of *Salmonella enterica* serotype Paratyphi A (*Salmonella* Paratyphi A) infections was reported in patients in Phnom Penh, Cambodia, and in European, American and Japanese travellers returning from Cambodia. Epidemiological investigations did not identify a common source of exposure. To analyse the population structure and genetic diversity of these *Salmonella* Paratyphi A isolates, we used whole-genome sequencing on 65 isolates collected from 1999 to 2014: 55 from infections acquired in Cambodia and 10 from infections acquired in other countries in Asia, Africa and Europe. Short-read sequences from 80 published genomes from around the world and from 13 published genomes associated with an outbreak in China were also included. Pulsed-field gel electrophoresis (PFGE) was performed on a subset of isolates. Genomic analyses were found to provide much more accurate information for tracking the individual strains than PFGE. All but 2 of the 36 isolates acquired in Cambodia during 2013–2014 belonged to the same clade, C5, of lineage C. This clade has been isolated in Cambodia since at least 1999. The Chinese outbreak isolates belonged to a different clade (C4) and were resistant to nalidixic acid, whereas the Cambodian outbreak isolates displayed pan-susceptibility to antibiotics. Since 2014, the total number of cases has decreased, but there has been an increase in the frequency with which nalidixic acid-resistant C5 isolates are isolated. The frequency of these isolates should be monitored over time, because they display decreased susceptibility to ciprofloxacin, the first-choice antibiotic for treating paratyphoid fever.

## Data Summary

All supporting data, code and protocols have been provided within the article or through supplementary data files. The whole-genome sequence reads from the *Salmonella* Paratyphi A used in this study have been deposited in the European Nucleotide Archive. The run accession numbers and related metadata are detailed in Table S1 (available in the online Supplementary Material), which has been deposited in FigShare: DOI:10.6084/m9.figshare.346180.

(https://figshare.com/articles/Genomic_analysis_of_Salmonella_enterica_serotype_Paratyphi_A_during_an_outbreak_in_Cambodia_2013-2015/3464180).

## Impact Statement

The bacterium *Salmonella* Paratyphi A can cause bloodstream infections (paratyphoid fever) and is transmitted via food or water contaminated with waste products from infected individuals. Accurate genetic information about bacterial isolates is crucial to support public health investigations, for tracing transmission and identifying the source, in particular. Classical typing methods, such as pulsed-field gel electrophoresis (PFGE), do not perform well on this genetically monomorphic bacterial population. We used whole-genome sequencing and comparative genomics for the retrospective investigation of an outbreak of *Salmonella* Paratyphi A infections in Cambodia. The greater discriminatory power and portability of genomic analyses, compared with PFGE, made it possible to identify the isolates associated with the outbreak unambiguously, and to assess their genetic relationships with isolates from sporadic cases, historical isolates and isolates from another outbreak in China.

## Introduction

*Salmonella enterica* serotypes Typhi (*Salmonella* Typhi) and Paratyphi A (*Salmonella* Paratyphi A) are Gram-negative bacteria confined to human hosts. They can invade the bloodstream, causing typhoid and paratyphoid fever (known together as ‘enteric fever’). These infections have become rare in Western countries, but they continue to be responsible for a considerable burden of disease in low-resource settings (Bhan *et al.*, 2005). Historically, enteric fever was mostly caused by *Salmonella* Typhi. However, the number of *Salmonella* Paratyphi A infections has been increasing steadily over the last 20 years, and it is now estimated that there are six million cases of paratyphoid fever annually (Newton *et al.*, 2016). This increase is most apparent in Asia, where *Salmonella* Paratyphi A now accounts for 14 % (Indonesia) to 64 % (South-East China) of enteric fever cases (Ochiai *et al.*, 2005). Furthermore, international travellers returning from countries in which *Salmonella* Paratyphi A is endemic are increasingly being infected with this bacterium, which accounted for 31 % of imported cases of enteric fever in the European Union in 2011 (ECDC, 2013).

During 2011–2013, an unusual increase in the number of *Salmonella* Paratyphi A infections was reported among patients attending the Sihanouk Hospital Centre of HOPE (SHCH), Phnom Penh, Cambodia (Vlieghe *et al.*, 2013). This increase coincided with an obse﻿rved increase in the number of cases of *Salmonella* Paratyphi A infection among travellers returning to Europe, the USA, New Zealand or Japan from Cambodia (Tourdjman *et al.*, 2013; Saitoh *et al.*, 2016; Judd *et al.*, 2015). No similar increase in *Salmonella* Typhi infections was reported. Epidemiological investigations identified no common source of exposure and the route of disease transmission remained unknown. The analysis of population structure and genetic diversity among outbreak isolates is an important component of epidemiological investigations. Such information can provide clues to the origin and transmission of the bacterial pathogens. Molecular epidemiology analysis was carried out on only 22 *Salmonella* Paratyphi A isolates from Japanese travellers infected during 2012–2014 (Saitoh *et al.*, 2016). PFGE with a single enzyme (*Xba*I) identified a predominant profile (18/22, 81.8 %) among the outbreak isolates, suggesting that the outbreak was caused by a single strain. However, PFGE, the previous gold standard for subtyping *Salmonella* spp., has proved unsuitable for the analysis of highly clonal serotypes like *Salmonella* Paratyphi A (Tien *et al.*, 2011). In this study, our main aim was to use accurate genetic information, from whole-genome sequencing data in particular, to analyse the population structure and genetic diversity of the *Salmonella* Paratyphi A isolates collected during the 2013–2015 outbreak in Cambodia.

## Methods

### Surveillance systems – Cambodia.

The Cambodian isolates were collected at the SHCH, a 40-bed non-governmental referral hospital for adults in Phnom Penh. Since July 2007, SHCH and the Institute of Tropical Medicine in Antwerp, Belgium, have been jointly organising the surveillance of bloodstream infections at this hospital. Between 2008 and 2015, 190 *Salmonella* Paratyphi A and 64 *Salmonella* Typhi isolates (considering 1 isolate per patient) were obtained from a total of 18 917 blood cultures.

### Surveillance systems – France.

In France, typhoid and paratyphoid fever have been notifiable diseases since 1903. Physicians and clinical laboratories are responsible for the mandatory reporting of cases. Clinical laboratories also send isolates to the French National Reference Centre for *Escherichia coli*, *Shigella* and *Salmonella* (FNRC-ESS), Institut Pasteur, Paris, on a voluntary basis. For each isolate, epidemiological data for the patient, including the date and site of isolation, sex and age, are recorded, together with the patient’s history of international travel. During the 2000s, the FNRC-ESS network included a stable number of hospital and private clinical laboratories referring about 65 % of all human *Salmonella* isolates identified in France to the FNRC-ESS (Le Hello *et al.*, 2013). From 2008 to 2015, 85 430 serotyped *Salmonella* isolates were registered at the FNRC-ESS.

For paratyphoid fever, we merged data from both the FNRC-ESS and the notifiable diseases surveillance system, and identified duplicates. From 2008 to 2015, 311 cases of *Salmonella* Paratyphi A infection were reported to both the notifiable diseases surveillance system and the FNRC-ESS. These included 50 cases of *Salmonella* Paratyphi A infection in patients with a history of travel to Cambodia. Isolates were available from the FNRC-ESS for 47 of these cases (94 %).

For typhoid fever, only data from the FNRC-ESS were analysed, as legal constraints precluded duplicate identification. From 2008 to 2015, 1162 *Salmonella* Typhi isolates were received at the FNRC-ESS. Seven of these isolates were obtained from patients reporting a history of travel to Cambodia.

### Bacterial isolates.

The 65 *Salmonella* Paratyphi A isolates sequenced for this study are listed in Table S1 (available in the Supplementary Material). They originated from the collections of the FNRC-ESS (*n*=31), the SHCH (*n*=30), and the Enteric and *Leptospira* Reference Laboratory at the Institute of Environmental Science and Research Limited, Wallaceville, New Zealand (*n*=4). The isolates were obtained from blood (*n*=59), stools (*n*=4), urine (*n*=1) or other sources (*n*=1). Conventional methods and serotyping at the FNRC-ESS, as previously described (Grimont & Weill, 2007), confirmed that all 65 isolates were *Salmonella* Paratyphi A.

### Antibiotic-susceptibility testing.

Antibiotic susceptibility was determined by disc diffusion on Mueller–Hinton agar, in accordance with the guidelines of the Antibiogram Committee of the French Society for Microbiology (CA-SFM & EUCAST, 2014). The following 32 antimicrobial drugs (Bio-Rad) were tested: ampicillin, ticarcillin, piperacillin, piperacillin/tazobactam, cefamandole, cefoperazone, cefoxitin, cefotaxime, amoxicillin/clavulanic acid, ticarcillin/clavulanic acid, imipenem, meropenem, ertapenem, cefepime, ceftazidime, streptomycin, spectinomycin, kanamycin, amikacin, gentamicin, netilmicin, tigecycline, isepamicin, nalidixic acid, pefloxacin, ciprofloxacin, sulfonamides, trimethoprim, sulfamethoxazole/trimethoprim, chloramphenicol, tetracycline and azithromycin. Minimal inhibitory concentration (MIC) values for nalidixic acid, ciprofloxacin and azithromycin were determined by Etests (bioMérieux). *E. coli* CIP 76.24 (ATCC 25922) was used as a control.

### Identification of *gyrA* mutations associated with decreased susceptibility to ciprofloxacin.

The quinolone-resistance-determining region (QRDR) of the *gyrA* gene encoding the DNA gyrase A subunit was sequenced in five nalidixic acid-resistant *Salmonella* Paratyphi A isolates obtained from travellers returning from Cambodia during 2014–2015, as previously described (Weill *et al.*, 2006). The nucleotide and deduced amino acid sequences were analysed and compared with sequences available from the National Center for Biotechnology Information website (NCBI – http://www.ncbi.nlm.nih.gov/ – last accessed 16 May 2016).

### PFGE.

PulseNet standard PFGE of *Xba*I-digested chromosomal DNA was carried out for a selection of 51 *Salmonella* Paratyphi A isolates (Ribot *et al.*, 2006). *Bln*I-PFGE was also used to type a subsample of 24 isolates.

### Single-nucleotide polymorphism (SNP) genotyping assay.

Three informative SNPs were selected from the 4810 SNPs identified in the comparative genomic analysis, for the reliable identification of lineage A, and clades C2 and C5 of lineage C. For lineage A, the specific SNP (A to G at position 666 468 of the *Salmonella* Paratyphi A ATCC 9150 reference genome, GenBank accession no. NC_006511) was within the SPA_RS02955 gene. For lineage C, the clade C2-specific SNP (G to T at position 4 294 741) was within the SPA_RS20855 gene, whereas the C5-specific SNP (G to A at position 2 381 607) was within the SPA_RS11495 gene. Three pairs of primers were designed to amplify fragments containing the three SNPs: forward primer cladeA-F (5′-TCC GAG TAG CAT TGG CTT GC-3′, ATCC 9150 co-ordinates 666 181–666 200) and reverse primer cladeA-R (5′-GAG CAG CCG CCT GAA TCA AC-3′, co-ordinates 666 755–666 736); forward primer cladeC2-F (5′-CGG AAA CTG ATG GAC TCA GC-3′, co-ordinates 4 294 490–4 294 509) and reverse primer cladeC2-R (5′-TCG ACA TAA GTC CCG TCA GC-3′, co-ordinates 4 294 992–4 294 973); and forward primer cladeC5-F (5′-CCG TTA ATC GTT GCC GTA GC-3′, co-ordinates 2 381 338–2 381 357) and reverse primer cladeC5-R (5′-CAA CGA TGC CGT TGA GTT GG-3′, co-ordinates 2 381 851–2 381 832). High-purity salt-free oligonucleotides were obtained from Eurofins Genomics. Total DNA was extracted with the InstaGene matrix kit (Bio-Rad), in accordance with the manufacturer’s recommendations. PCRs were performed in a 50 µl reaction volume containing MgCl_2_ (1.5 mM), deoxynucleotide triphosphates (each at 0.1 mM), GoTaq Flexi DNA polymerase (0.85 U) and its buffer (Promega), dimethyl sulfoxide (5 %), 10 pmol each primer and 2 µl template DNA. Amplification began with a first denaturation step at 94 °C; followed by 35 cycles of denaturation for 30 s at 94 °C, annealing for 30 s at 57 °C for C2 and C5 and 58 °C for A, polymerisation for 1 min at 72 °C; and a final extension step at 72 °C for 10 min. Sanger DNA sequencing of the PCR products was carried out with a Big Dye Terminator V3.1 cycle sequencing kit (Applied Biosystems) and a 96-capillary 3730xl DNA Analyzer (Applied Biosystems), by Eurofins MWG Operon. The nucleotide sequences were analysed with Bionumerics v.6.6 software (Applied-Maths). The blastn program of NCBI (http://www.ncbi.nlm.nih.gov/ – last accessed 16 May 2016) was used for SNP identification.

### Whole-genome sequencing.

High-throughput genome sequencing was carried out at the genomics platform of the Pasteur Institute, on the HiSeq2500 platform (Illumina) generating 138 to 151 bp paired-end reads, yielding a mean of 169-fold coverage (minimum 74-fold, maximum 407-fold).

### Other genomes studied.

A selection of 93 published *Salmonella* Paratyphi A short-read sequences (Zhou *et al.*, 2014; Yan *et al.*, 2015) were downloaded from the European Nucleotide Archive (EMNL-EBI – http://www.ebi.ac.uk/ena – last accessed May 16 2016) and included in this study. These sequences were selected from high-quality short-read sequences, to cover the broadest range of time, geographical origin and genomic diversity for published *Salmonella* Paratyphi A isolates. We included 80 of the 149 genomes from the study by Zhou *et al.* (2014) and 13 of the 22 genomes from the study by Yan *et al.* (2015). The accession numbers of each of these genomes are listed in Table S1. The genomes of the *S. enterica* serotypes Choleraesuis (GenBank accession no. AE017220), Dublin (CP001144), Enteritidis (AM933172), Gallinarum (AM933173), Heidelberg (CP001120), Newport (CP001113), Agona (CP001138), Paratyphi B (CP000886), Paratyphi C (CP000857), Schwarzengrund (CP001127), Typhi (AE014613) and Typhimurium (AE006468) were used as outgroups.

### Read alignment and SNP detection.

For each isolate, we aligned the paired-end reads with the *Salmonella* Paratyphi A ATCC 9150 reference genome (McClelland *et al.*, 2004) (GenBank accession no. NC_006511), using Bowtie with default parameters (Langmead & Salzberg, 2012). SAMtools (Li *et al.*, 2009) was then used to build a genome index and to identify SNPs from the Bowtie alignments. Several criteria were used to filter the resulting SNPs: a minimum coverage (number of reads mapped to the reference genome) of 20 and a minimum quality score of 25 for each SNP. The SNPs retained were concatenated to generate a multiple alignment of all SNPs by an in-house Perl script. The resulting sequences were further filtered to remove all SNPs present in insertion sequences identified by ISfinder (Siguier *et al.*, 2006). Other repetitive regions were identified by a self-self blast analysis (Altschul *et al.*, 1990) of the reference sequence, using the following parameters: megablast (word size 28), identity percentage >95 % and match length >400 bp. This masked 3.3 % of the reference genome. Finally, clusters of SNPs introduced via horizontal sequence transfer were detected and removed with Gubbins (Croucher *et al.*, 2015).

### Phylogenetic and temporal analyses.

SNP alignments were analysed with Bayesian evolutionary analysis sampling trees (BEAST) (Drummond & Rambaut, 2007) version 1.8.2, which was used to reconstruct temporal phylogenetic trees and to estimate divergence times. The input consisted of a continuous alignment of 4810 non-repetitive, non-recombinant SNPs from 159 strains, together with the numbers of invariant A, C, T and G nucleotides. The concatenated SNP alignments were subjected to multiple BEAST analyses with both constant-size and Bayesian skyline population size-change models, in combination with strict, log-normal or exponential relaxed clock models, to identify the best-fit model. For the BEAST analysis, the GTR+Γ substitution model was selected, and tip dates were defined as the year of isolation. For all model combinations, three independent chains of 100 million generations each were run to ensure convergence, with sampling every 1000 iterations. Convergence and effective sample size values were inspected with Tracer (Drummond & Rambaut, 2007) version 1.5. A marginal likelihood estimation was carried out, with path sampling and stepping stone sampling for each run that had converged, to compare the different combinations of clock and tree models (Baele *et al.*, 2012, 2013). We then used marginal likelihood estimation to determine which model gave the best fit, by calculating the Bayes factor. The relaxed (uncorrelated exponential) clock model, which allows evolutionary rates to vary between the branches of the tree, and the skyline demographic model provided the best fits to the data (Table S2), as previously reported by Zhou *et al.* (2014).

The parameter and tree estimates of the three runs were combined with LogCombiner (Drummond & Rambaut, 2007) version 1.7.5, with the first 20 % of states in each chain removed as burn-in. Maximum clade credibility trees were generated with TreeAnnotator (Drummond & Rambaut, 2007) version 1.7.5 on the combined files, and visualised with FigTree version 1.4.2 (Drummond & Rambaut, 2007).

Furthermore, a maximum-likelihood (ML) approach with mega6 (Tamura *et al.*, 2013) was used to support the Bayesian phylogeny. For the ML analysis, mega6 was run with the general time-reversible model and a gamma distribution, to model site-specific rate variation (i.e. the GTR+Γ substitution model). One hundred bootstrap replicate analyses were performed to assess the ML phylogeny. The final tree was visualised in FigTree version 1.4.2.

### Genetic analyses.

The presence and type of antibiotic-resistance genes (ARGs) were determined with ResFinder version 2.1 (Zankari *et al.*, 2012). The presence of mutations in the QRDR of the DNA gyrase and topoisomerase IV genes was assessed by the visual examination of *de novo* assembled sequences.

### Pan-genome analysis.

Roary (Page *et al.*, 2015) version 3.2.4 was used on SPAdes (Bankevich *et al.*, 2012) assemblies annotated with Prokka (Seemann, 2014) to construct a pan-genome.

## Results

### Epidemic curves

**Fig. 1.**
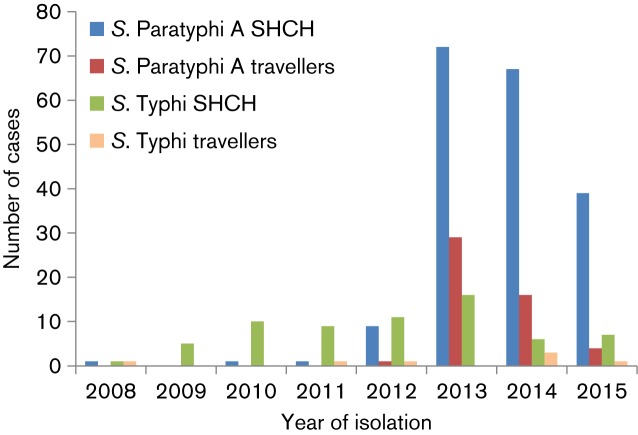
Enteric fever cases at SHCH, Phnom Penh, Cambodia, and in travellers returning to France from Cambodia, 2008–2015. The numbers of blood culture-confirmed cases of enteric fever (only the first isolate per patient was included) at SHCH and the numbers of confirmed cases in migrants or travellers returning to France from Cambodia during the 2008–2015 period are shown.

During the 2008–2012 period, enteric fever in patients attending the SHCH and in travellers returning from Cambodia to France was caused mostly by *Salmonella* Typhi (Fig. 1). During this period, 12 cases of *Salmonella* Paratyphi A infection were identified among patients attending the SHCH, and only 1 such case was reported in a traveller returning from Cambodia to France. In 2013, a sharp increase in the number of cases was observed both at the SHCH and among travellers returning from Cambodia to France, with a total of 72 and 29 cases, respectively. In 2014 and 2015, the annual number of cases decreased, but remained higher than for the period preceding 2013. During this outbreak, the number of *Salmonella* Typhi cases remained relatively stable.

In total, during the 2013–2015 period, 178 *Salmonella* Paratyphi A cases were reported among SHCH patients, and 49 cases were reported among travellers returning from Cambodia to France. The median age of the affected patients at the SHCH during this period was 26 years (interquartile range 22–31). Male and female patients were equally affected (48.9 % of the cases were male); none of these patients died. The median age of the affected travellers returning to France was 33 years (interquartile range 27–49), and 43 % were male; none of these patients died.

### Phylogenetic and temporal analyses

We performed short-read genomic sequencing (Illumina) on 65 *Salmonella* Paratyphi A isolates (Table S1), including 55 from patients in or returning from Cambodia, obtained between 1999 and 2014. Thirty of these fifty-five Cambodian isolates were collected in Phnom Penh, between 2008 and 2013, the other 25 being collected from travellers returning to France or New Zealand from Cambodia (including seven who had also travelled to neighbouring countries, such as Vietnam, Laos, Thailand and Malaysia) or migrants from Cambodia. The other ten isolates sequenced were obtained between 2013 and 2014, from French travellers who had not visited Cambodia but had travelled to other countries in South-East Asia, Southern Asia or Africa, and from a case where the patient had not travelled abroad (laboratory contamination). Short-read sequences from 80 published *Salmonella* Paratyphi A genomes (Zhou *et al.*, 2014) were selected and included in our study, to provide the population structure framework of this serotype, with its seven lineages, A to G. Short-read sequences from 13 published *Salmonella* Paratyphi A genomes associated with a large-scale community outbreak in Southern China during the 2010–2011 period were also included (Yan *et al.*, 2015). The assembled genome of ATCC 9150 – a laboratory strain of unknown origin (McClelland *et al.*, 2004) – was used as the reference genome. The genomic analysis was carried out on a final set of 159 *Salmonella* Paratyphi A genomes, including 63 linked to Cambodia during the 1958–2014 period.

**Fig. 2.**
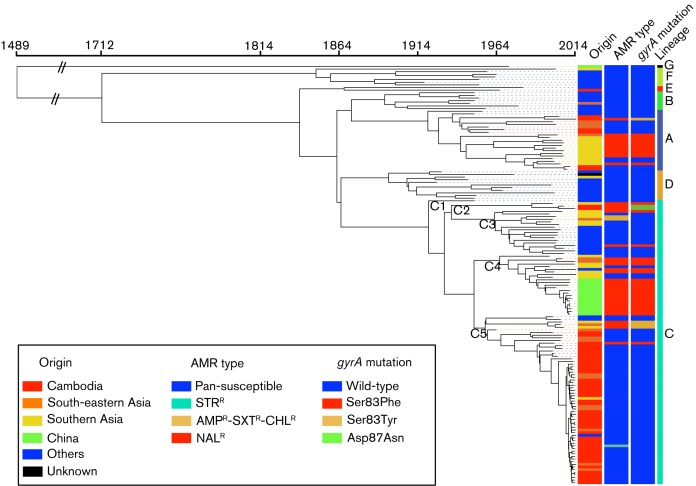
Timed phylogeny for 4810 SNPs in 159 *Salmonella* Paratyphi A genomes. A maximum clade credibility tree generated by BEAST 1.8 (exponential clock rate and Bayesian skyline population models) is shown, providing information about the geographical origin, antimicrobial resistance phenotype (AMR type), *gyrA* mutation encoding resistance to nalidixic acid and lineages of the genomes. The different clades (C1 to C5) of lineage C are also indicated. The tips of the tree are coloured according to the geographical origin of the genomes. The abbreviations used for the antibiotics are as follows: AMP, ampicillin; NAL, nalidixic acid; STR, streptomycin; SXT, trimethoprim/sulfamethoxazole. AMP^R^ indicates resistance to AMP. NAL^R^ indicates resistance to NAL, which was associated with decreased susceptibility to ciprofloxacin (MIC in the range 0.125–1 mg l^−1^).

ML (Fig. S1) and BEAST (Fig. 2) phylogenetic analyses were performed on 4810 chromosomal SNPs from the non-repetitive non-recombinant core genome of 159 *Salmonella* Paratyphi A genomes (Table S3). Both approaches confirmed the presence of the seven lineages, A to G, described by Zhou *et al.* (2014). Three lineages, A (*n*=7, 1958–2014), E (*n*=1, 1965) and C (*n*=55, 1999–2014) were observed among the 63 genomes (55 from our study and 8 from that of Zhou *et al.*, 2014) acquired in Cambodia between 1958 and 2014. Lineage C could be subdivided into five clades, C1 to C5, with all but 2 of the 55 Cambodian lineage C isolates belonging to C5. The two remaining isolates belonged to C2.

ML and BEAST analyses revealed that all but 3 of the 47 isolates collected in Cambodia or obtained from travellers returning from Cambodia between 2010 anin clade C5 (Table 1 and S1, Figs 2 and S1). During the 2013–2014 outbreak period, all but 2 of the 36 isolates acquired in Cambodia belonged to clade C5. The two remaining isolates belonged to genetic groups A and C2. Certain SNPs were identified exclusively within clade C5. Thirty-two of these specific SNPs were found in more than 70 % of the sixty-four C5 isolates, including three found in all the C5 isolates (Table S4). The mean intra-clade pairwise SNP variation within C5 isolates was 23 (minimum 1–maximum 82), whereas it was 7 (1–29) if only the C5 isolates collected between 2010 and 2014 were taken into account. No particular geographical or temporal clustering was observed for the patients hospitalised at the SHCH and infected with such C5 isolates. This outbreak strain was derived from a strain that had been circulating locally and regionally, as C5 isolates had already been isolated in Cambodia from 1999, and in Vietnam from 1963 to 2005 (Table S1). The C5 strain responsible for the outbreak was also isolated from travellers returning from Nepal (one case), Thailand (one case) and Vietnam (one case) in 2013 (Table S1, Fig. S1). However, the Cambodian outbreak strain was different from that responsible for the Chinese outbreak in 2010–2011 (Yan *et al.*, 2015), which belonged to clade C4 (Table 2, Table S1, and Fig. 2). The C4 Chinese isolates differed from the C5 isolates by 77 SNPs on average (48–91), and the mean intra-clade pairwise SNP variation within C4 isolates was 11 (3–33).

**Table 1.** Characteristics of the 63 *Salmonella* Paratyphi A genomes linked to Cambodia, 1958–2014 The number of isolates (*n*) is indicated when *n*>1; it includes seven isolates from patients who had also travelled to neighbouring countries, such as Vietnam, Laos, Thailand and Malaysia. AMR type, Antimicrobial resistance type; NAL, nalidixic acid; STR, streptomycin.

Period	AMR type (*n*)	Lineage (*n*)	No. of genomes
1958–1998	Pan-susceptible (3)	A (2), E	3
1999–2009	Pan-susceptible (10)	C5 (8), A (2)	13
	NAL (3)	A*, C2†, C5*	
2010–2014	Pan-susceptible (44)	C5 (43), A	47
	NAL (2)	A‡, C2†	
	STR	C5	

*Ser83Phe *gyrA* mutation encoding NAL resistance.

†Asp87Asn *gyrA* mutation encoding NAL resistance.

‡Ser83Tyr *gyrA* mutation encoding NAL resistance.

**Table 2.** Characteristics of the 55 Asian *Salmonella* Paratyphi A genomes not linked to Cambodia, 1943–2014 The number of isolates (*n*) is indicated when *n*>1. AMR type, Antimicrobial resistance type; MDR, multi-drug resistant (to ampicillin, streptomycin, sulfamethoxazole-trimethoprim, chloramphenicol and tetracycline); NAL, nalidixic acid; STR, streptomycin.

Period	Southern Asia (*n*=28)		South-East Asia (*n*=12)		Eastern Asia (*n*=15)	
	**AMR type (*n*)**	**Lineage (*n*)**	**AMR type (*n*)**	**Lineage (*n*)**	**AMR type (*n*)**	**Lineage (*n*)**
1943–1998	Pan-susceptible (5)	C4 (2), C3 (2), D	Pan-susceptible (3)	A, B, C5^1^	Pan-susceptible	G
	NAL	A*				
1999–2009	Pan-susceptible (5)	A (2), C3, C4, F	Pan-susceptible (3)	A (2), C5	NAL (3)	C4* (3)
	NAL (13)	A* (8), C5† (2), C2*, C3*, C4*	NAL (2)	C4*, C5†		
	MDR	C3	MDR	C3		
2010–2014	Pan-susceptible (2)	C4, C5	Pan-susceptible (2)	C5 (2)	NAL (11)	C4* (11)
	NAL	C4*	NAL	C4*		

1 Oldest C5 genome (Vietnam, 1963).

*Ser83Phe *gyrA* mutation encoding NAL resistance.

†Ser83Tyr *gyrA* mutation encoding NAL resistance.

**Fig. 3.**
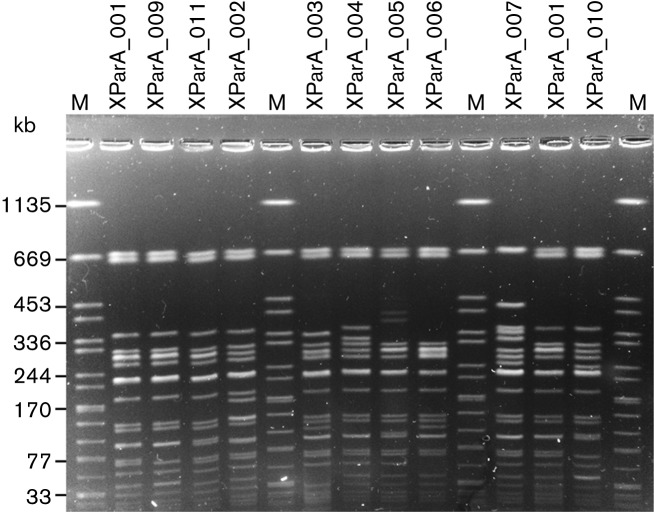
Representative *Xba*I-PFGE profiles of *Salmonella* Paratyphi A isolates. The 10 different *Xba*I–PFGE profiles obtained from the analysis of 51 *Salmonella* Paratyphi A isolates are shown. Lanes 1, 6, 11 and 15, *S. enterica* serotype Braenderup H9812 used as a molecular size marker (M) (band sizes in kb); lane 2, C5 isolate 4416 (XParaA_001); lane 3, C3 isolate 201300701 (XParaA_009); lane 4, C5 isolate 201404185 (XParaA_011); lane 5, C4 isolate 201304008 (XParaA_002); lane 7, A isolate 03–2557 (XParaA_003); lane 8, C4 isolate 201301308 (XParaA_004); lane 9, C5 isolate 201311858 (XParaA_005); lane 10, A isolate 201400552 (XParaA_006); lane 12, C4 isolate 201403926 (XParaA_007); lane 13, C5 isolate 99–7427 (XParaA_001); and lane 14, C5 isolate 5288/46 (XParaA_010).

We also assessed the genetic diversity of the *Salmonella* Paratyphi A isolates by PulseNet standardized PFGE. A subsample of 51 *Salmonella* Paratyphi A isolates was first analysed by *Xba*I-PFGE (Table 3 and Fig. 3). A predominant profile, XParA_001 was observed in 35/41 (85.4 %) C5 isolates analysed. However, three other *Xba*I-PFGE profiles were seen in the remaining C5 isolates. The XParA_001 profile was also found in 75 % (3/4) of the C2 and C3 isolates analysed, but not in C4 isolates, which were phylogenetically closer to C5 (Fig. 2). Similar findings were obtained for analyses with a second enzyme, *Bln*I, on 24 of the 51 isolates (Fig. S2). A predominant profile, BParA_001, was observed in 14/15 C5 isolates analysed. However, another profile was seen in the remaining Cambodian outbreak C5 isolate. The BParA_001 profile was also found in all other C2 and C3 isolates tested, but not in C4 isolates.

**Table 3.** Correlation between PFGE data and genomic sequences from 51 *Salmonella* Paratyphi A isolates

Lineage	Combined *Xba*I-/*Bln*I-PFGE profiles (*n*)*	No. of genomes
A	XParA_003/BParA_003, XParA_006/BParA_006	2
C2	XParA_001/BParA_001, XParA_001/nd	2
C3	XParA_001/BParA_001, XParA_009/BParA_001	2
C4	XParA_004/BParA_004 (2), XParA_002/BParA_004, XParA_007/BParA_007	4
C5	XParA_001/nd (24), XParA_001/BParA_001 (11), XParA_011/BParA_001 (2), XParA_011/nd (2), XParA_005/BParA_001, XParA_010/BParA_002	41

nd, Not determined.

*The number of isolates (*n*) is indicated when *n* >1.

### Antibiotic-susceptibility testing and determination of resistance mechanisms

The Cambodian outbreak strain was pan-susceptible to antibiotics, with the exception of one isolate that was resistant only to streptomycin. This outbreak strain was susceptible to quinolones (i.e. nalidixic acid) and fluoroquinolones (i.e. ciprofloxacin), in particular. By contrast, the two non-C5 isolates collected during the outbreak in Cambodia were resistant to nalidixic acid (MIC>256 mg l^−1^) with decreased susceptibility to ciprofloxacin (MIC 0.09–0.38 mg l^−1^). This resistance was mediated principally by mutations in the QRDR of *gyrA*, the chromosomal DNA gyrase A gene. We therefore checked for such mutations within this QRDR. We identified mutations leading to a serine to tyrosine substitution at codon 83 (Ser83Tyr) in the lineage A isolate from 2014, and to an aspartic acid to asparagine substitution at codon 87 (Asp87Arg) in the clade C2 isolate from 2013. Quinolone-resistant *Salmonella* Paratyphi A bacterial populations were also present in Cambodia before the outbreak period, as we identified three nalidixic acid-resistant strains isolated there during the 2000–2006 period. They belonged to lineage A, and clades C2 and C5 of lineage C, and they carried the *gyrA* mutations Ser83Phe, Asp87Asn and Ser83Phe, respectively (Table 1). The C5 nalidixic-acid resistant isolate was collected in 2000, 11 years before the outbreak. The Chinese outbreak was also caused by a strain resistant to nalidixic acid with decreased susceptibility to ciprofloxacin (Yan *et al.*, 2015). Within the genomic sequences of the Chinese isolates, we identified the very common *gyrA* mutation Ser83Phe (Table 2, Table S1). Overall, various *gyrA* mutations were acquired at least 10 times in the 159 global *Salmonella* Paratyphi A genomes studied, but only in lineages A and C (Fig. 2). The *gyrA* mutation leading to Ser83Phe substitution was the most frequently observed.

### Pan-genome analysis

The pan-genome analysis (Fig. S3) carried out by Roary identified a total of 7009 genes, including 3342 for the core genome, in the 159 genomes studied. We detected no gains of particular accessory genes nor losses of common genes in the Cambodian outbreak strain (Table S5).

### Recent evolution in Cambodia

The epidemic curve based on FNRC-ESS data showed a sharp decrease in the number of *Salmonella* Paratyphi A infections among travellers returning to France from Cambodia between 2013 (*n*=25) and 2015 (*n*=5). However, the number of nalidixic acid-resistant isolates increased from 0 % (0/25) in 2013 to 12.5 % (2/16) in 2014 and 80 % (4/5) in 2015. The only nalidixic acid-resistant isolate sequenced here, 201400552, belonged to lineage A and had the *gyrA* mutation leading to a Ser83Tyr substitution. The question was, therefore, whether the other five recent nalidixic acid-resistant isolates belonged to lineage A or to the outbreak C5 clade, with the acquisition of a mutation affecting the QRDR of *gyrA*. As these five isolates were collected very recently (after the whole-genome study), we developed a SNP genotyping assay to determine whether these isolates belonged to lineage A or clades C2 and C5 of lineage C, which are known to contain nalidixic acid-resistant isolates. In this SNP assay, the five recent nalidixic acid-resistant isolates were found to belong to the C5 clade and to have the *gyrA* mutation leading to the Ser83Phe substitution.

## Discussion

The analysis of whole-genome sequencing data from isolates unambiguously identified a single predominant strain (C5 clade) among the isolates collected during the recent outbreak of paratyphoid fever in Cambodia, and made it possible to assess the phylogenetic relationships between this strain and other strains circulating in Cambodia or elsewhere in Asia at or before this time. The inclusion of many other publicly available genomes, including, in particular, 80 representative genomes from a global collection of 149 *Salmonella* Paratyphi A isolates obtained between 1917 and 2009, increased the discriminatory power of the genomic epidemiology analysis, making it possible to define and trace back the outbreak strain.

The genomic analysis showed that the C5 clade was not confined to Cambodia, but was also found in travellers returning from Nepal, Thailand and Vietnam in 2013, suggesting that the outbreak strain may have been circulating beyond the borders of Cambodia. However, these countries did not experience similar outbreaks of *Salmonella* Paratyphi A infections during the same period. An outbreak of *Salmonella* Paratyphi A infections was reported in China in 2010–2011, but we found that this outbreak was caused by a strain from a different clade, C4. However, the C4 and C5 clades, separated by a mean of 77 SNPs, shared a common ancestor around 1950.

By contrast, PFGE, the widely-used gold standard method for subtyping *Salmonella* spp. to the strain level, did not attain the same discriminatory power, despite the presence of a predominant PFGE profile among the outbreak isolates. The presence of other PFGE profiles in the C5 outbreak isolates made the epidemiological analysis harder to interpret. For example, the 11 C5 isolates collected at the SHCH in Phnom Penh during 2012–2013 presented three different PFGE profiles (the main profile, XParA_001, in seven isolates, XParA_011 in three isolates and XParA_010 in one isolate). Furthermore, the predominant *Xba*I- and *Bln*I-PFGE profiles, XParA_001 and BParA_001, were also observed in C3 isolates from West Africa. In addition to the weak discriminatory power of this low-throughput fingerprinting method, the PFGE data had low portability. Despite using the same standardized protocol, the figures showing PFGE data in the papers by Saitoh *et al.* (2016) and Yan *et al.* (2015) do not contain a molecular size ladder, precluding any comparative analysis between these isolates and our isolates. By contrast, the increasing availability in public databases of short-read sequences from published genomes or of sequences generated during the routine surveillance of *Salmonella* infections (Ashton *et al.*, 2016) has strongly improved the identification and investigation of outbreaks, particularly those of an international nature.

Unlike the strain responsible for the Chinese outbreak in 2010–2011, the Cambodian outbreak strain was pan-susceptible to the antibiotics tested. This antibiotic susceptibility is of particular importance, as a clear trend towards the acquisition of antibiotic resistance in *Salmonella* Paratyphi A isolates from Asia has been observed, despite the lack of data from South-East Asia (Gu *et al.*, 2015; Teh *et al.*, 2014). The susceptibility of the outbreak strain to quinolones, and the isolation of a nalidixic acid-resistant C5 strain in Cambodia 11 years before the current outbreak, suggest that neither quinolone resistance nor resistance to any other antibiotic was an important bacterial factor in this outbreak, at least before 2014. Despite a decreasing number of cases between 2013 and 2015, both at the SHCH and in travellers returning from Cambodia to France (Fig. 1), the increase in the isolation of nalidixic acid-resistant C5 isolates from 2014 is worrying, as these isolates also had decreased susceptibility to ciprofloxacin, the first-line treatment in adults. We now need to monitor *Salmonella* Paratyphi A infections and their susceptibility to antibiotics to determine whether there is a real trend towards an increase in nalidixic acid-resistant strains over time. These recent C5 isolates are also being investigated by whole-genome sequencing, to determine whether they form a tight cluster or are distributed evenly throughout the C5 clade, and whether they are derived from the 2013–2014 outbreak strain or from more ancestral C5 populations.

In their comprehensive comparative genomics analysis of a global collection representative of the population structure of this serotype, Zhou and co-workers concluded that the crucial genomic elements of *Salmonella* Paratyphi A responsible for causing paratyphoid fever were already present in the most common recent ancestor dating from the mid-15th century. Furthermore, they found that almost all genetic changes (gene acquisitions or losses and mutations) since that time had been transient (i.e. removed by purifying selection) (Zhou *et al.*, 2014). These findings are indicative that environmental and/or human behavioural factors enhancing transmission to naive hosts are more likely to explain outbreaks of paratyphoid fever than the recent emergence of a particularly virulent *Salmonella* Paratyphi A strain. Such environmental and/or human behavioural risk factors for paratyphoid fever have been investigated in case–control studies in Asia. In Nepal, different routes of infection were identified for *Salmonella* Typhi and *Salmonella* Paratyphi (Karkey *et al.*, 2013). The significant identified risk factors for *Salmonella* Paratyphi A infections were the consumption of street food during the 2 weeks preceding the onset of illness and residence in the capital city, Kathmandu, for less than 2 years. In Indonesia, *Salmonella* Typhi and *Salmonella* Paratyphi A were also found to follow different routes of infection (Vollaard *et al.*, 2004). The risk factors associated with a *Salmonella* Paratyphi A infection were identified as the consumption of food from street vendors and flooding. In South China, during a community outbreak of 600 cases, the main risk factor was the consumption of uncooked vegetables from street stalls, local markets or small restaurants (Yan *et al.*, 2015). The primary source was a vegetable patch irrigated with untreated hospital wastewater.

These findings highlight the need for solid public-health investigations and interventions, together with food-safety regulations, in Cambodia. Significant steps in terms of food safety were taken in Cambodia in 2015, when a draft of the country’s first food law was released.

For future public health investigations, the use of whole-genome sequencing data in real-time, together with classical epidemiological methods, would be advantageous, as demonstrated here by the unequalled discriminatory power of genomic data for tracking individual strains (i.e. distinguishing outbreak-associated isolates from those of contemporary sporadic cases and assessing the true genetic relationships between isolates) from this genetically monomorphic bacterial population. The collection of epidemiological and molecular data should be a collaborative effort, to maximise the gain from combining the two types of data. Efforts should also be made, during initial investigations, to isolate strains from suspected water and/or food sources and from identified carriers. This should make it possible to optimise the use of high resolution provided by whole-genome sequencing and to demonstrate a clear link with the suspected source, as demonstrated by Yan *et al.* (2015) during the outbreak in China in 2010–2011.

## Supplementary Data

1005 Supplementary File 1

6871 Supplementary File 2
