# Supplementary figures and images for: Genomic analysis of Salmonella enterica serotype Paratyphi A during an outbreak in Cambodia, 2013–2015
Source: Microb Genom. 2016 Nov 30;2(11):e000092. doi: 10.1099/mgen.0.000092 (PMC5320704; doi:10.1099/mgen.0.000092)

FEB  
A  
D  
C

Lineage

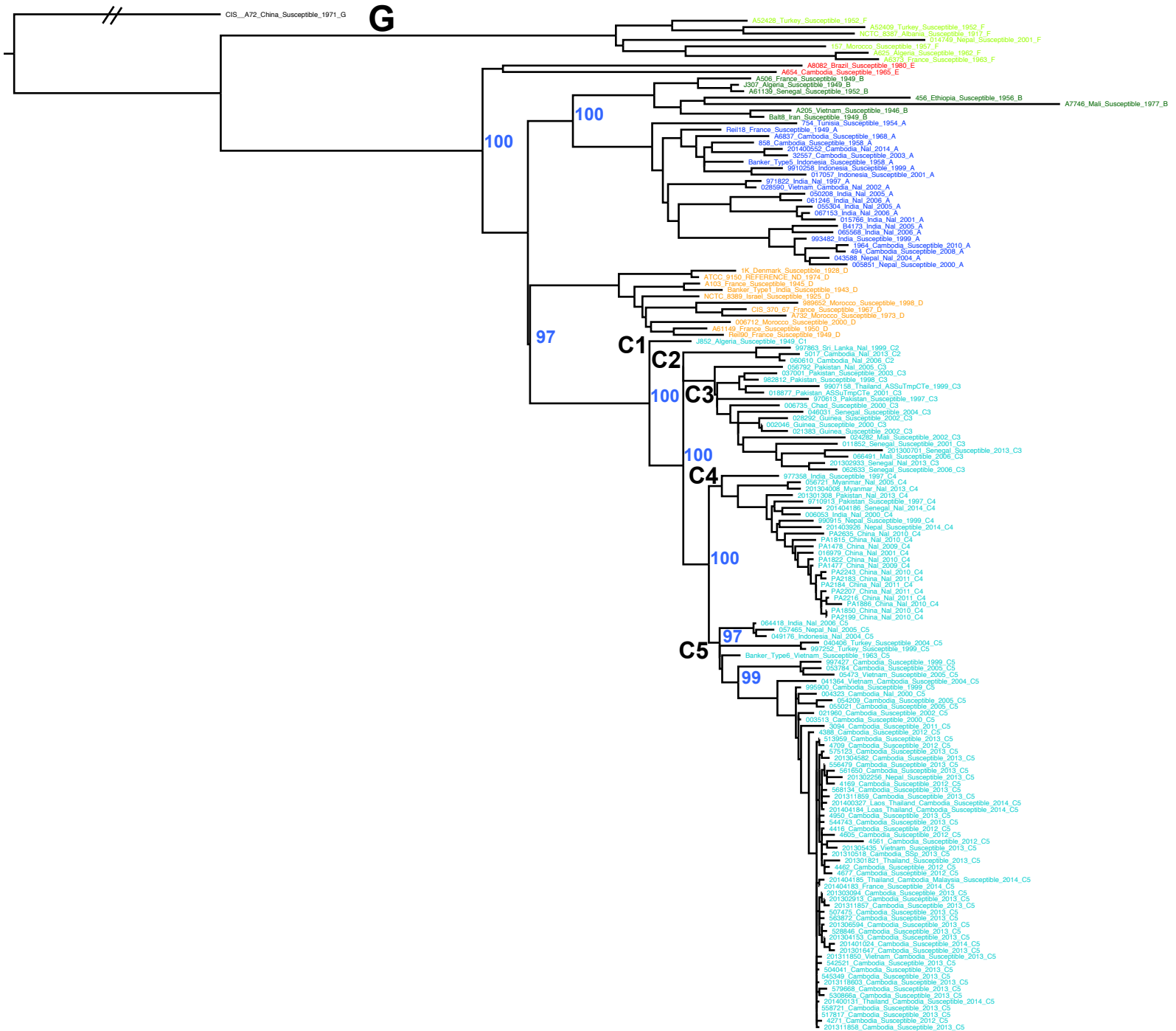

Supplementary Figure 1

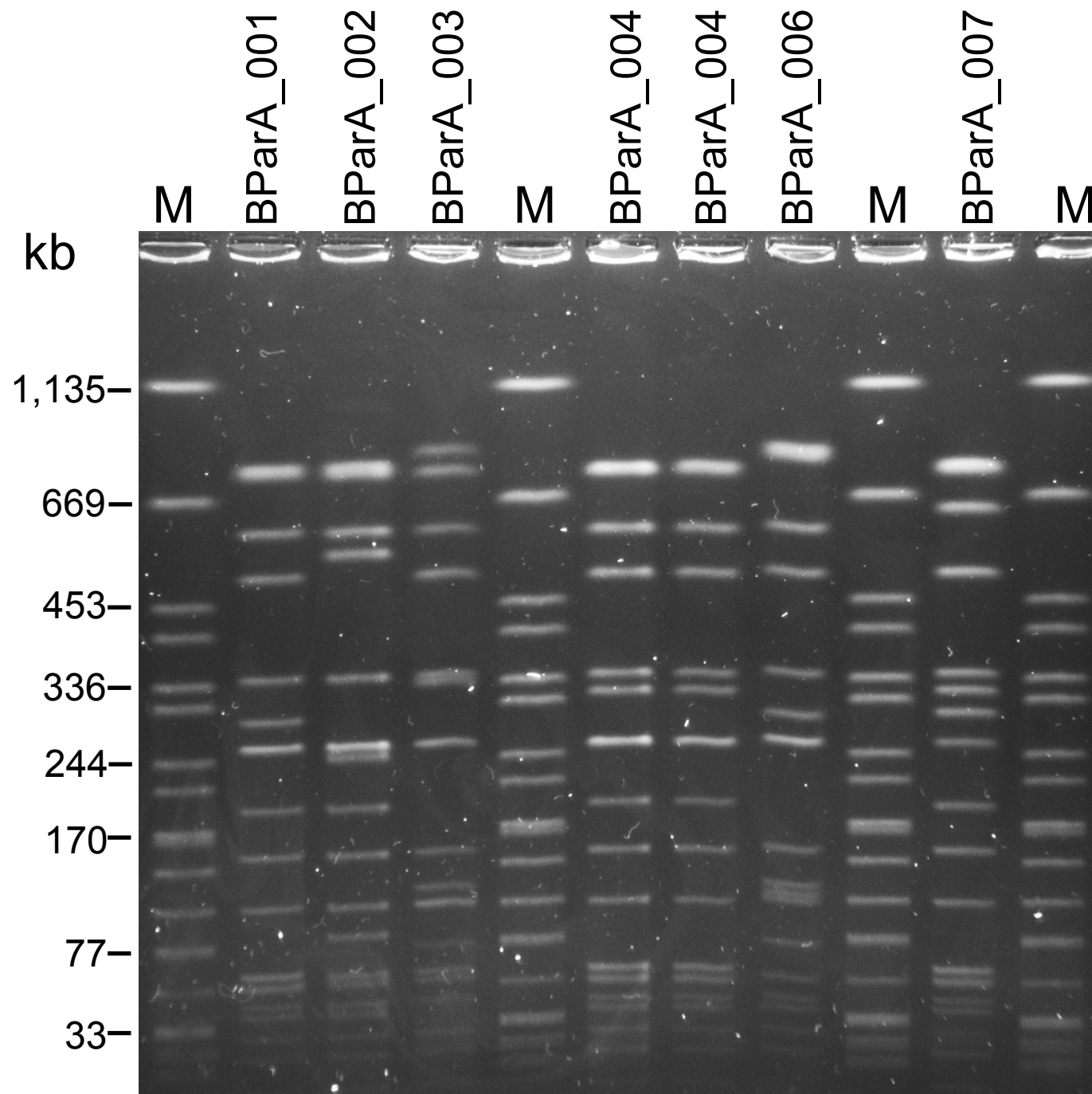

Supplementary Figure 2

a

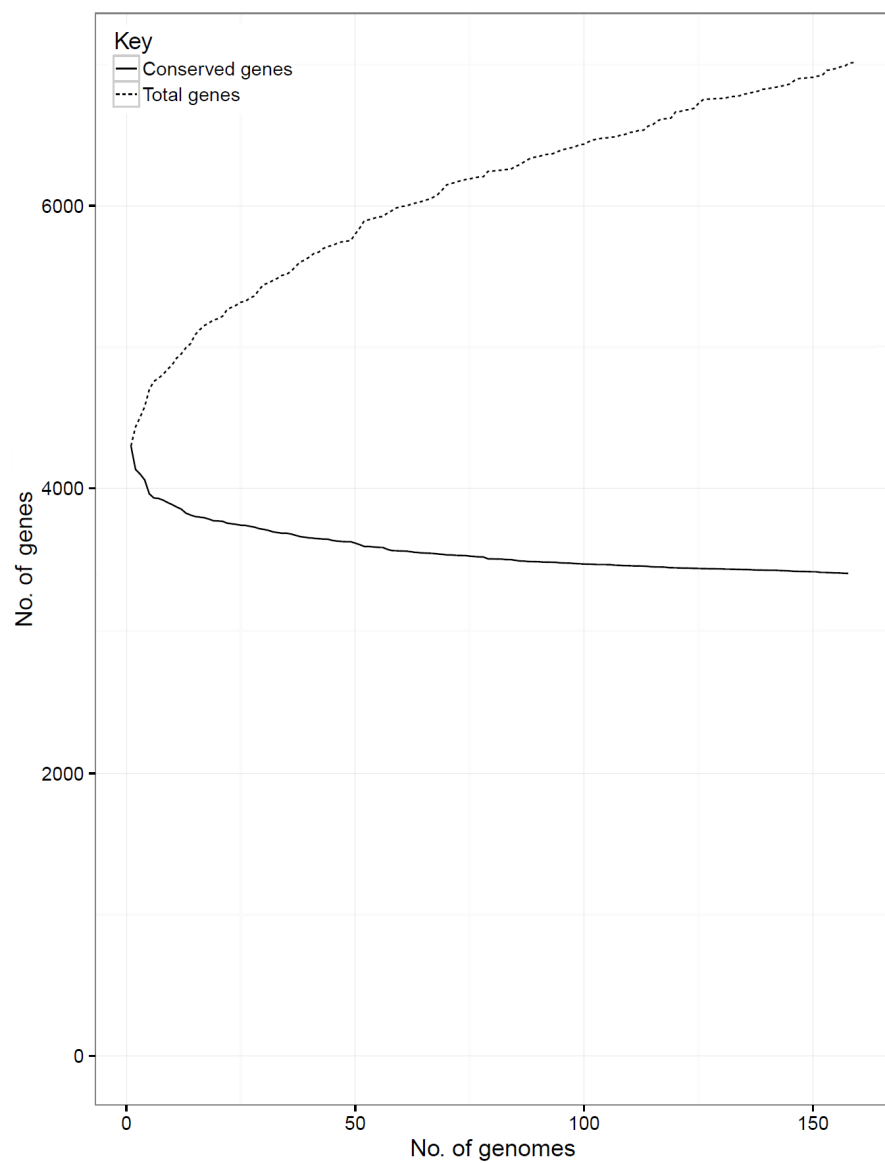

b

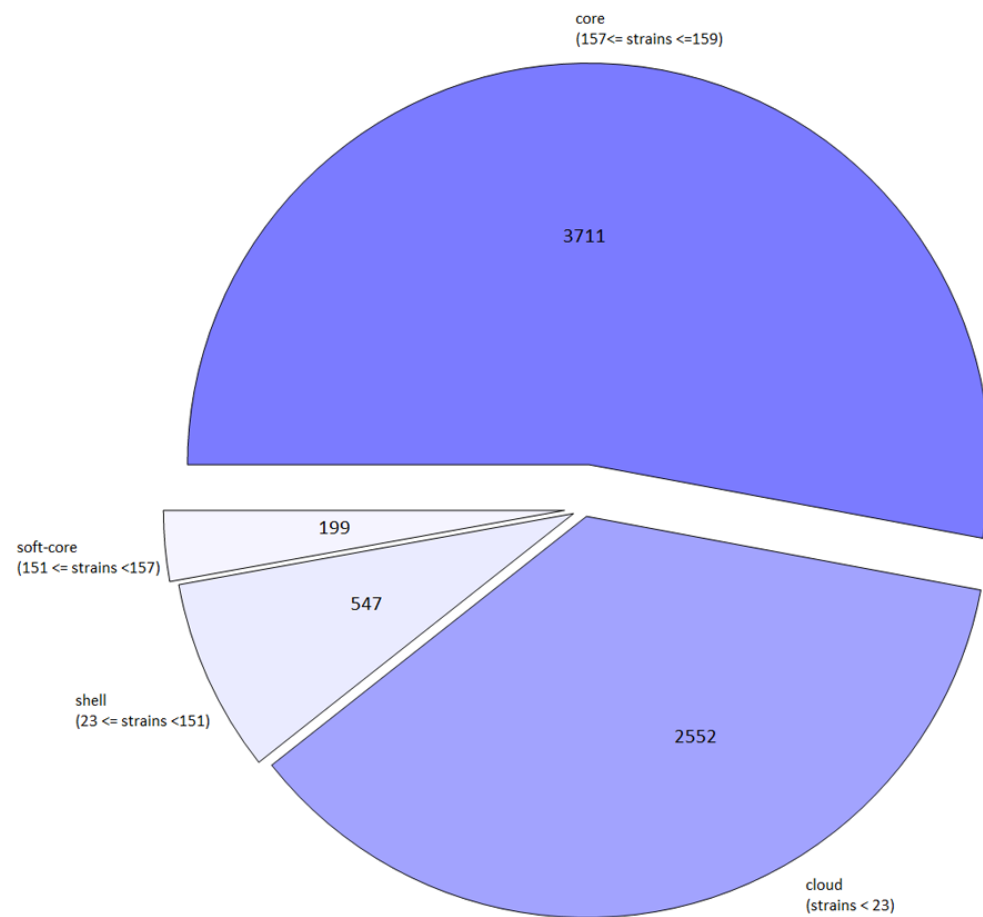

Supplementary Figure 3

Supplement: Supplementary File 1 [file mgen-02-92-s001.pdf]
